# Prominent Asymmetric Muscle Weakness and Atrophy in Seronegative Immune-Mediated Necrotizing Myopathy

**DOI:** 10.3390/diagnostics11112064

**Published:** 2021-11-08

**Authors:** Sunha Park, Dae-Hyun Jang, Jae-Min Kim, Nara Yoon

**Affiliations:** 1 Department of Rehabilitation Medicine, Incheon St. Mary’s Hospital, College of Medicine, The Catholic University of Korea, Seoul 06591, Korea; juddypark5@gmail.com (S.P.); dhjangmd@naver.com (D.-H.J.); 2 Department of Pathology, Incheon St. Mary’s Hospital, College of Medicine, The Catholic University of Korea, Seoul 06591, Korea; waxggul@gmail.com

**Keywords:** immune-mediated necrotizing myopathy, inflammatory myopathy, asymmetric muscle involvement

## Abstract

Immune-mediated necrotizing myopathy, a new subgroup of inflammatory myopathies, usually begins with subacute onset of symmetrical proximal muscle weakness. A 35-year-old male presented with severe asymmetric iliopsoas atrophy and low back pain with a previous history of left lower extremity weakness. Although his first left lower extremity weakness occurred 12 years ago, he did not receive a clear diagnosis. Magnetic resonance imaging of both thigh muscles showed muscle edema and contrast enhancement in patch patterns, and the left buttock and thigh muscles were more atrophied compared to the right side. Serum creatine kinase levels were elevated, and serologic testings were all negative. Genetic testing using a targeted gene-sequencing panel for neuromuscular disease including myopathy identified no pathogenic variants. Muscle biopsy on the right vastus lateralis showed scattered myofiber necrosis with phagocytosis and an absence of prominent inflammatory cells, consistent with seronegative necrotizing myopathy. Thus, unusual asymmetric muscle weakness and atrophy can be a manifestation of inflammatory myopathy.

## 1. Introduction

Immune-mediated necrotizing myopathy (IMNM) is a new subgroup of inflammatory myopathies, distinguished by myositis-specific-antibodies (MSAs), including anti-signal recognition particles (anti-SRP) and anti-3-hydroxy-3-methylglutaryl coenzyme A reductase (anti-HMGCR) [1,2,3]. The discovery of these two MSAs has resulted in the identification of two subgroups of IMNM patients with distinct clinical-serum profiles. In a recent study, seronegative IMNM formed a distinctive subgroup of IMNM [4].

Electromyography and serologic testing are commonly performed in patients with proximal muscle weakness and elevated creatinine phosphokinase (CPK) levels. Negative serological findings, along with electromyography suggesting myopathy, can cause confusion in diagnosis. A patient’s clinical features may be nonspecific, and accurate pathological diagnosis and genetic testing play an important role in diagnosis. We present a case in which a seronegative patient with histology-proven IMNM developed prominent asymmetric proximal muscle atrophy in the stepwise subacute disease course.

## 2. Case Presentation

**Figure 1:**
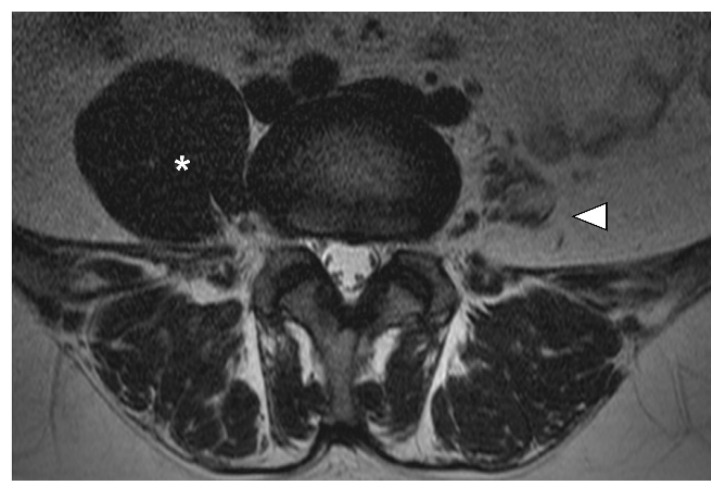
Magnetic resonance imaging (MRI) of the lumbar region. Left iliopsoas muscle atrophy (white arrowhead) compared to the right side (star point) observed on lumbar MRI at the time of the initial visit.

A 35-year-old man was referred to the neuromuscular disease clinic of our institution from a local clinic due to severe left iliopsoas atrophy. He visited the local spine clinic due to a recent weakness in the left proximal leg and lower back pain. Lumbar magnetic resonance imaging (MRI) was performed and showed severe left iliopsoas atrophy but no definite spinal lesions (Figure 1). He denied any other medical history, acute infections, trauma or intoxication. In addition, there was no evidence of family history of muscle diseases. A detailed history indicated that the first left proximal lower extremity weakness event occurred 12 years ago. At that time, he underwent an electromyography at another clinic and the results suggested myopathy. A muscle biopsy was then performed on the left thigh muscle and the results were consistent with muscular dystrophy. Although he did not receive a clear diagnosis, his symptoms improved after taking prednisolone for about one year and, consequently, his medication was discontinued. Three years later, his left proximal muscle weakness reappeared, and he restarted prednisolone for six months and stopped again after symptoms improved. After that, he continued with his daily routine without any other symptoms.

**Figure 2:**
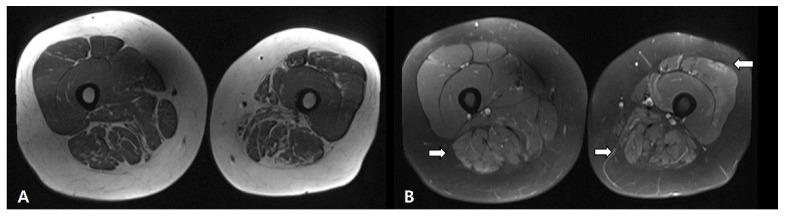
Results of magnetic resonance imaging (MRI) T1-dissusion (**A**) and T1-fat suppression (**B**) images of both thighs demonstrated diffuse high signal intensities on both thigh muscles (white arrows) and atrophy in the left buttock and thigh muscles.

When he visited our clinic, an initial physical examination revealed proximal motor weakness in the left leg, especially in the left hip flexion and adduction strength, using the grade 2 Medical Research Council (MRC) scale. There was no weakness in other extremities. He had no sensory disturbance and other neurologic examinations were normal. The laboratory examination showed an elevated CPK 4254 IU/L and AST/ALT 76/103 IU/L levels. Myositis-specific-antibodies including anti-SRP and anti-HMGCR were negative upon serologic testing. Anti-tRNA synthetase antibodies such as anti-Jo-1, non-specific myositis-associated antibodies such as anti-RNP antibodies and anti-SSA/SSB (Ro/La) were all negative. An MRI of both thigh muscles indicated that the left buttock and thigh muscles were more atrophied compared to the right side, and muscle edema and contrast enhancement were observed in patch patterns in both thigh muscles (Figure 2). Malignancy screening including computed tomography on the chest and abdomen did not suggest any evidence of concurrent malignancy. Nerve conduction studies were normal, and needle electromyography showed an early recruitment pattern, short duration and small amplitudes of motor unit action potentials, which were indicative of myopathy involving both lower extremities (Table 1). Genetic testing was performed using a targeted gene-sequencing panel for neuromuscular diseases that can assess 410 genes, including 136 genes associated with myopathy, and pathogenic variants were not detected [5].

**Figure 3:**
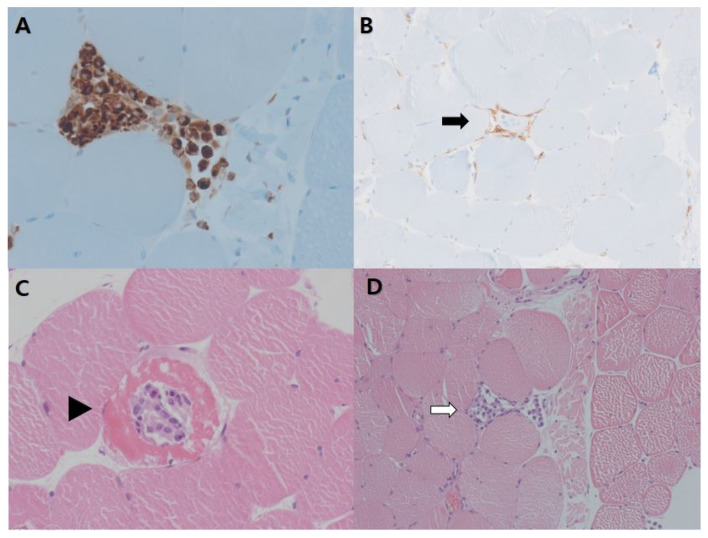
Muscle biopsy from the right vastus lateralis (**A**) Necrotic tissue infiltrated by macrophages (immunohistochemistry, CD68 stain) (×400) (**B**) Scant endomysial infiltration of CD4+ T-lymphocytes (arrows) (immunohistochemistry, CD4 stain) (×200) (**C**) Scattered necrotic myofibers (arrowhead) (×400) (**D**) with phagocytosis (white arrows) (high power field, Hematoxylin and eosin stain) (×200).

Muscle biopsy on the right vastus lateralis was performed and showed myofiber necrosis with phagocytosis and absence of prominent inflammatory cells, consistent with necrotizing myopathy (Figure 3). A diagnosis of seronegative IMNM was made based on the findings noted above. After receiving high-dose oral prednisolone (125 mg/day) and methotrexate (10 mg/week) treatment, the CPK levels gradually decreased to 259 U/L, and the left proximal limb muscle strength improved to a grade 3+ or 4 on the MRC scale. The patient is currently on an outpatient follow-up plan and tapering on medication.

## 3. Discussion

IMNM has been characterized by subacute onset of symmetrical proximal muscle weakness, elevated CPK levels, and myopathic findings of electromyography [3,6]. In a recent clinical study [7], 134 patients with inflammatory myopathy were reviewed, and only 13 patients (9.2%) with asymmetric muscle involvement were identified, which indicated that the condition is rare. The condition primarily presented with dermatomyositis and presented as IMNM in four patients. In this case report, muscle involvement was sub-acutely presented in a stepwise pattern over 12 years and was finally diagnosed as seronegative IMNM. At the onset of the first symptoms, the patient underwent a muscle biopsy, but no definitive diagnosis was made. Therefore, if a patient shows asymmetric muscle involvement and myopathy is suspected, it is important to perform a muscle biopsy with consideration of inflammatory myositis to achieve an accurate pathologic diagnosis.

The patient was diagnosed with antibody-negative IMNM, as he displayed proximal muscle weakness, increased muscle enzyme levels, myopathic-patterned electromyogram, no myositis-specific autoantibodies and muscle biopsies showing scattered necrotic fibers with macrophage predominant, paucilymphocytic infiltrates [2,3]. Some inherited disorders such as limb-girdle muscular dystrophy 2L (LGMD2L), dysferlinopathy (LGMD2B), and sarcoglycanopathies can show necrotic myofibers and CD8+ lymphocytic infiltrates, which can mimic IMNM [2]. Therefore, in the present case, genetic testing was performed using a targeted gene-sequencing panel for inherited muscular diseases, including *ANO5* (LGMD2L), *DYSF* (LGMD2B), *SGCA* (LGMD2D), *SGCB* (LGMD2E), *SGCD* (LGMD2F), *SGCE* (myoclonus-dystonia syndrome), and *SGCG* (LGMD2C), and no pathogenic variant was detected. Therefore, it is important to analyze the pathological findings and differential diagnosis of genetic diseases when the patient has atypical IMNM findings and is seronegative.

Seronegative IMNM was characterized in 2011, and it is difficult to estimate its current prevalence and incidence [1]. MSAs including anti-HMGCR and anti-SRP cannot be detected in a third of IMNM patients [2]. This subgroup has not yet been characterized in detail, except for an association with cancer [8]. Studies on the seronegative IMNM group are limited to the European population [4]. Clinical features exhibited by the Korean patient would be helpful to characterize the seronegative IMNM group. It should be noted that the case of one patient was seronegative, but IMNM was proven using histology. The subsequently developed anti-HMGCR antibodies later in the disease course, with an associated aggressive trajectory of clinical and biochemical deterioration [9]. Therefore, the possibility that tests could be seronegative due to a relatively low frequency of autoantibodies or the technical limitations of the applied laboratory tests cannot be excluded.

## 4. Conclusions

This case report describes a clinical feature of a stepwise subacute course of prominent asymmetric muscle weakness and atrophy, which was ultimately diagnosed as seronegative IMNM. Therefore, physicians should pay attention to unusual asymmetric muscle weakness or atrophy, which can be a manifestation of inflammatory myopathies. When the patient has atypical IMNM findings and is seronegative, it is important to analyze the pathological findings and differential diagnosis of inherited muscular diseases.

## Figures and Tables

**Table 1:** Findings of needle electromyography.

	Spontaneous Activities			Motor Unit Action Potential			Recruitment
	IA	Fib	PSW	Polyphasic	Amplitude	Duration	
R. L-PSP (L5)	N	1+	1+				
R. L-PSP (S1)	Decr	None	None				
L. L-PSP (L5)	N	1+	1+				
R. Biceps	N	None	None	N	N	N	N
R. FCR	N	None	None	N	N	N	N
R. FDI	N	None	None	N	N	N	N
R. G-max	N	None	None	N	N	N	N
R. Iliopsoas	N	None	None	N	Small	Short	N
R. V-med	N	None	None	N	N	N	N
R. TA	N	None	None	N	N	N	N
L. G-max	Decr	None	None	N	N	N	N
L. Iliopsoas	Decr	None	None	N	Small	Short	N
L. TFL	Decr	None	None	N	N	N	N
L. RF	Decr	2+	2+	N	Small	Short	Early
L. V-med	Decr	None	None	N	Small	Short	Early

IA: insertional activity, Fib: fibrillation potential, PSW: positive sharp wave, R: right, L: left, N: normal, Decr: decreased, L-PSP: lumbar paraspinal, FCR: flexor carpi radialis, FDI: first dorsal interosseous, G-max: gluteus maximus, V-med: vastus medialis, TA: tibialis anterior, TFL: tensor fasciae latae, RF: rectus femoris.

## Data Availability

Not applicable.
